# Supplementary material for: The SARS-CoV-2 Integrated Genomic Epidemiology Database (IGED): Linking viral genomes with patient-level metadata to advance statewide genomic surveillance in California
Source: PLOS Digit Health. 2026 Aug 31;5(8):e0001473. doi: 10.1371/journal.pdig.0001473 (PMC13528992; doi:10.1371/journal.pdig.0001473)
Supplement: Peer review history

# Peer review: Decision Letter 0

**DOI:** 10.1371/journal.pdig.0001473.r001

**Authors:** Hanieh Razzaghi

4 Aug 2026

The SARS-CoV-2 Integrated Genomic Epidemiology Database (IGED): Linking viral genomes with patient-level metadata to advance statewide genomic surveillance in California

PDIG-D-26-00825

Dear Dr. Wadford,

We are pleased to inform you that your manuscript 'The SARS-CoV-2 Integrated Genomic Epidemiology Database (IGED): Linking viral genomes with patient-level metadata to advance statewide genomic surveillance in California' has been provisionally accepted for publication in PLOS Digital Health.

Before your manuscript can be formally accepted you will need to complete some formatting changes, which you will receive in a follow-up email from a member of our team.

Please note that your manuscript will not be scheduled for publication until you have made the required changes, so a swift response is appreciated.

IMPORTANT: The editorial review process is now complete. PLOS will only permit corrections to spelling, formatting or significant scientific errors from this point onwards. Requests for major changes, or any which affect the scientific understanding of your work, will cause delays to the publication date of your manuscript.

If your institution or institutions have a press office, please notify them about your upcoming paper to help maximize its impact. If they'll be preparing press materials, please inform our press team as soon as possible -- no later than 48 hours after receiving the formal acceptance. Your manuscript will remain under strict press embargo until 2 pm Eastern Time on the date of publication. For more information, please contact digitalhealth@plos.org.

Thank you again for supporting Open Access publishing; we are looking forward to publishing your work in PLOS Digital Health.

Best regards,

Hanieh Razzaghi

Section Editor

PLOS Digital Health

***********************************************************

**Additional Editor Comments (if provided):**

**Reviewer Comments (if any, and for reference):**

Reviewer's Responses to Questions

**Comments to the Author**

1. Does this manuscript meet PLOS Digital Health’s publication criteria?

Reviewer #1: Yes

Reviewer #2: Yes

Reviewer #3: Yes

**********

2. Has the statistical analysis been performed appropriately and rigorously?

Reviewer #1: N/A

Reviewer #2: N/A

Reviewer #3: Yes

**********

3. Have the authors made all data underlying the findings in their manuscript fully available (please refer to the Data Availability Statement at the start of the manuscript PDF file)?

The PLOS Data policy

Reviewer #1: Yes

Reviewer #2: Yes

Reviewer #3: Yes

**********

4. Is the manuscript presented in an intelligible fashion and written in standard English?

Reviewer #1: Yes

Reviewer #2: Yes

Reviewer #3: Yes

**********

Reviewer #1: This manuscript is a thorough and well-written description of the IGED digital platform, including its interconnection with various other SC2 and public health platforms, linking PHI/PII, deidentified sequencing data, and reporting/presentation infrastructure in a tractable format. The authors describe the considerations and challenges in performing this kind of data collection and integration, and some hard-won approaches for resolving them in high-throughput real-world settings. It is clearly written and a valuable retrospective on the covid pandemic with lessons for future pandemic preparedness and ongoing public health genomic epidemiology.

Reviewer #2: Thank you to the authors for this interesting and important work. The manuscript was very thorough and did a good job of describing the rigorous methods undertaken to create and utilize the linked dataset. I appreciate how much work went into standardizing the submissions from multiple sources and continuously monitoring the data quality. I have no major comments at this time.

Reviewer #3: I appreciate the authors' efforts in conducting this research and preparing the manuscript. Overall, the article is well-presented, and the organization of its structure and content is highly systematic and logical.

**********

what does this mean?). If published, this will include your full peer review and any attached files.

**Do you want your identity to be public for this peer review?**  If you choose “no”, your identity will remain anonymous but your review may still be made public.

For information about this choice, including consent withdrawal, please see our Privacy Policy

Reviewer #1: No

Reviewer #2: No

Reviewer #3: **Yes:**  Tahmina Akhter

**********
